# Marital satisfaction through the lens of Iranian women: a qualitative study

**DOI:** 10.11604/pamj.2016.25.208.9769

**Published:** 2016-12-05

**Authors:** Zeinab Tavakol, Zahra Behboodi Moghadam, Alireza Nikbakht Nasrabadi, Nikzad Iesazadeh, Maryam Esmaeili

**Affiliations:** 1 Department of Reproductive Health, School of Nursing and Midwifery, Tehran University of Medical Sciences, Tehran, Iran; 2 Department of Midwifery, School of Nursing and Midwifery, Shahrekord University of Medical Sciences, Shahrekord, Iran; 3 Department of Medical-Surgical Nursing, School of Nursing and Midwifery, Tehran University of Medical Sciences, Tehran, Iran; 4 Department of Research Center of the Quran, Hadith and Medicine, Tehran University of Medical Sciences, Tehran, Iran; 5 Critical Care Department, School of Nursing and Midwifery, Nursing and Midwifery Care Research Center, Tehran University of Medical Sciences, Tehran, Iran

**Keywords:** Marital, personal satisfaction, content analysis, Iranian women

## Abstract

**Introduction:**

One of the common concepts to show the happiness and stability of marriage is the concept of marital satisfaction. Marital satisfaction plays an important role in the stability of marriage. This study was conducted to explain the perception of marital satisfaction among Iranian women.

**Methods:**

This study was conducted between March and September 2015 by common qualitative content analysis approach through semi-structured interviews and 19 participants were selected by purposive sampling.

**Results:**

With the analysis of data two themes: (maturity of personality) which included a sub-theme of blossoming of feelings, and (romantic interaction) consisted of three sub-themes of; mutual support, sense of peace and joyful dependence emerged.

**Conclusion:**

Marital life can lead to the development of people and lovely interaction between them. Surely it needs to passing of time and self-knowing and couple-knowing of each other. Family consultants need the perception of how couple's interaction is, also they need to understand about couples who can communicate well to each other so they can overcome many life's other deficiencies.

## Introduction

One of the common concepts to show the happiness and stability of marriage is the concept of marital satisfaction [1]. Marital satisfaction offers an overall assessment of the current situation among couples [2], and is the adjustment of current situation with the expected situation among them. Family psychology specialists' criteria in evaluating the quality of marital relationships are the level of marital satisfaction among couples. It´s one of the most important factors that affect progress, achievement of life goals and emotional stability of the couples [3, 4]. Research has shown that, couples who understand each other and feel satisfy with their relationship, have better performance and comply better with their roles [4]. From the perspective of Quran which is the Muslims' holy book and is considered as a complete and comprehensive guide for humanity, marriage has three goals: reproduction, sexual and non-sexual instincts satisfaction and peace [5]. Marital satisfaction is the feeling of happiness, satisfaction, and joy experienced by the husband or wife in all aspects of their marriage, and is an important part of personal health, especially mental health [6]. Because marital satisfaction has an effect on mental and physical health, life satisfaction, success in business and social connections, and as it is considered as a protective factor against psychological trauma caused by mishaps is life [7]. Marital satisfaction is something that requires couples to strive for it.

Marital satisfaction is a multidimensional concept that includes various factors such as personality attributes, finances, parenting styles and sexual relations [8, 9]. On the other hand, studies have shown that, marital satisfaction has several dimensions including; sexual satisfaction, receiving support from partner, partner's health, communication, personality attributes, relationships with others, participation in decision making and relationship with partner's family, leisure time, common religion and religious beliefs, and social support [9–13]. Many reports, despite their differences, show that, the prevalence of marital dissatisfaction in on the rise in different ways [14]. Although, in Iran due to religious beliefs, specific cultural fabric, and prevail ethical and moral values, the intensity of family problems is not as high as some other countries, the existence of dissatisfactions and problems in this area cannot be ignored [15]. In fact, in Iran, according to culture and religion, marriage and establishment of a successful family life is considered women's life accomplishments, and most Iranian single women consider themselves unsuccessful even if they have achieved many successes in the field of academic qualifications, employment and income.

In Iran, getting married and success in marriage is considered as one of the most important issues for women, and the unsuccessful marriage and getting divorced have undesirable consequences for them. However, according to official and unofficial statistics as well as the experiences of research team marriage in Iran shows a downward trend and number of divorces is increasing each year. It is noticeable that, majority of these divorces happen at the first three years of marital life (which show a decrease from the first five years to the first three years of marital life). In fact, marital lives are not stable and show a downward trend. In recent years, Iranian families and society have faced with many changes such as; increased age of the marriage, patterns of marriage, shortening of the fertility and childbearing period, reduction of intra-generation bonds, increased women's education and employment status, reduction of intimate relations and time together [16, 17].

Since the health and vitality of family members is one of the necessary conditions to achieve economic, social and cultural development, to strengthen family respect and stability requires an understanding of the factors influencing the growth and stability of the family, the authors have decided to explore the factors affecting marital satisfaction among Iranian women. Also because of the subjectivity of the topic, little knowledge, obvious need to look deep into this subject, and unresponsiveness of quantitative research with the positivism pattern to explain the meaning of marital satisfaction, thus the use of qualitative research methods of naturalism was necessary. Therefore, this study was conducted to explore marital satisfaction from the perspective of Iranian women, in order to provide a suitable guideline for young couples to develop and maintain a happy and stable marital life.

## Methods

**Study design:** To achieve this objective, a qualitative study based on conventional content analysis was used. Qualitative studies are used to increase understanding and describing the human experience [18]. To understand the complexities of social and psychological phenomena, qualitative methodologies are usually used. The value of qualitative methodologies to assess the psychological consequences of marriage is increasingly recognized. Content analysis is a systematic approach and coding system that can be used to explore a lot of background information to determine communication patterns and trends [19].

**Collection of information and participants:** in this study, participants were selected by purposive sampling method and maximum variation in length of marriage, parenthood status, education level, and employment status of women. Semi-structured interviews were conducted with 19 Persian speaking married women living in Tehran with the desire and ability to express their experiences of marital satisfaction. All women were interviewed in a private room without the presence of their spouses, and interviews were recorded with their permission. Each interview lasted around 55 minutes on average. Then implementation was carried out carefully. Questions were included as follow: 1) please talk about one day experience of your married life; 2) in what way you can be satisfied with your marital life? 3) What factors do you think are effective in increasing marital satisfaction? 4) What factors do you think are effective in reducing marital satisfaction?

All data were gathered from the beginning of 2014 winter until the end of 2015 summer. After giving information to qualified women about the study's aim, semi-structured interviews were conducted in relation to marital satisfaction. All interviews were conducted in Farsi at the beginning and then translated into English. The second author acted as a bilingual interpreter monitored and confirmed the translation. Each interview was recorded and transcribed verbatim and was analyzed simultaneously.

**Data analysis:** According Graneheim & Landman (2004), the following steps were taken to analyze the data: 1) the verbatim transcript of the interviews and reading several times to get the full concept; 2) Categorization of the text into compressed semantic units; 3) summarization of compressed semantic units and naming them with codes; 4) sorting codes into categories and sub-categories based on similarities and differences; 5)developing themes as internal text concept [20].

**Rigour:** This study examined different aspects of the reliability. Reliability was obtained through review by the participants, review by colleagues and prolonged interaction. Review by the participants was carried out by questioning the participants' responses to confirm transcripts and emerged codes from the interviews. Team members consulted each other in dealing with any ambiguity in the process of coding, classification and identification of themes. To address the portability, complete set of analyzed documentation and data are on the file and are available on demand.

**Ethical considerations:** This article is part of the first author's doctoral thesis. Tehran University of Medical Sciences Ethics Committee has approved the study's proposal. All participants were informed about the objectives and methodology of the study. To record interviews, permission was obtained from the participants. They knew that participation in this study is voluntary, and they can leave at any time without any negative impact on administrative services they receive. Those who agreed to participate in the study signed an informed consent form.

## Results

All participants´ demographics have shown in Table 1. With the analysis of data two themes: (maturity of personality) which included a sub-theme of blossoming of feelings, and (romantic interaction) consisted of three sub-themes of mutual support, sense of peace and joyful dependence emerged.

**Table 1:** Demographic traits of participant

No.	Age	Sex	Level Of Education	Job Status	Number Of Children	Year Of Marital Life
1	37	Woman	Ph.D	University Teacher	1	7
2	36	Woman	Student of Ph.D	Physician	2	12
3	44	Woman	Student of Ph.D	University Teacher	1	16
4	33	Woman	Student of Ph.D	Physician	1	6
5	43	Woman	Secondary School	Nurse Aid	2	27
6	40	Woman	Associate Degree	Employee	2	22
7	45	Woman	Student of Ph.D	University Teacher	2	22
8	28	Woman	Student of Ph.D	Housewife	0	3
9	28	Woman	Student of Ph.D	Housewife	0	3.5
10	24	Woman	Bachelor	Housewife	1	2
11	31	Woman	Diploma	Employee part time	1	5
12	50	Woman	Illiterate	Housewife	3	25
13	29	Woman	Diploma	Housewife	2	8
14	25	Woman	Diploma	Housewife	1	4.5
15	29	Woman	Diploma	Housewife	1	6
16	31	Woman	Diploma	Employee	1	3
17	37	Woman	Diploma	Housewife	2	20
18	32	Woman	Middle School	Housewife	2	12
19	36	Woman	Primary School	Housewife	2	22

### Maturity of personality

The maturity of personality theme included a sub-theme of blossoming of feelings. These feelings actually are created or strengthened during a person´s marital life, and contribute to the continuation of marital life. Sense of responsibility and commitment to each other is one of these feelings. Couples admit that, they have responsibilities in marital life and are obliged to comply with them. This sense of responsibility causes couples to be committed to marital life and do their best to solve family issues. This commitment encompasses couples' family life issues and also their economic, social and moral issues. From the perspective of these women, couples' commitment to marital relationship is the most important issues. For women who participated in this study, independent decision-making power of their husbands and the independence of their husbands from their parents were considered very important factors. They stated that, it is desirable that, man should be able to make decision about their life without the involvement of his family. In this regard, one participant who had been married for three years, stated: “It is very important that families do not interfere, not my parent, not his parent, nobody interfere, we ourselves decide what to do, we might ask them for advice, they may advise us what to do, what not to do, but, we finally decide what to do ourselves.” Sense of independence in decision-making is very important for couples. Because they think, they know their living conditions more than anybody else, and have the ability to make decisions for their lives.

Another feeling that matures and blossoms during the marriage is the sense of adjustment with current situation. Couples learn, in order to keep their life, need to adjust themselves with their spouses' and their families' attributes, and current situation. This adjustment comes about after knowing individuals and surrounding environment. However, it must be noted that, individuals' adjustment and compromise with their spouses' characteristics and existing situation develops over time. This can increase the ability of forgiveness. Forgiving some irrational personal wishes of partners could make living conditions better for them and causes positive feedback from them. In relation to this issue, one participant said, “For example, I like to do something that my partner does not like, I forbear once and my partner forbear once, this way we solve the problem.” On the other hand, being mother and achieve a sense of motherhood can affect one's satisfaction with his/her marriage and married life. According to one of the participants; “Well before my children were born I felt empty, now I feel that my life is perfect, there is no vacuum in my life anymore.” This feeling is very precious for most women and shows that, having a baby is one of the perfections of their lives. A woman, who becomes mother, has a deeper feeling towards her husband, and as the love of child is their common interest, while reducing the distance between them, increases their love and affection, especially when they have an agreement over child rearing and parenting style. Both are trying to overcome their child´s needs, and modify their living conditions to provide a better future for their child.

### Romantic interaction

The next theme is romantic interaction that includes the three sub-themes of mutual support, sense of peace and joyful dependence.

#### Mutual support

According to the participants' statements, when women see their husbands as a supporter and protector they have more marital satisfaction. According to one participant, this feeling is like: *“I feel that he is always behind me, in the worst situations, I am confident that, there is someone at home who is ready to face difficulties for me, he has done things that I never thought he would do, just for me.”* In fact, couples are close people to each other, so when problems occur, they are the first person to get their support. Almost all participants talked about the effect of partner's cooperation in homework and childcare, and believed that, partner's help and cooperation can help them in education and even make them less tired and as a result make them a happier person. For instance, one of the participants said: *“My husband helps me with homework, for example; he washes the dishes, especially when we have guests, I am responsible for cooking, and he does some dusting and vacuuming. This has a big impact on my satisfaction.”*

Accompany and ongoing support of partner can help to maintain self-esteem in women. However, marital satisfaction in woman, who does not receive support by her husband when a problem arises or get blamed, will be affected. One participant also was upset about this issue and said: *“When I make a mistake, my husband is quick to blame me and this gives me a feeling as if I am an ignorant while I am not like that”.*

#### Sense of peace

In this approach, to love and be loved is the most obvious sense. Couples, who induce the sense of loving towards each other will be more satisfied with their marital life. This feeling is constantly changing and evolving among couples; early in their life it is more thrilling and shallow and over time becomes deeper and wiser. Couples can provide romantic love satisfaction for each other in life by expressing their feelings honestly and clearly toward each other. Expressing feeling was one of the issues raised by most participants. However most women were dissatisfied with the lack of expression of feelings by their husbands and recognized that as a negative factor in many future problems of their relationships. One of the participants in this regard, said: *“I love my husband to put his feeling in word and say it, he says I love you in my heart, I say no, people must express their love, I do not know what is in your heart? I want him to say his feeling than keep it in his heart.”*

Respecting each other is also among factors that affect couples' marital satisfaction. The importance of this issue is beyond the range of the couple´s relationship and affects their relationship with their partners' families. It is noteworthy that, this sense of appreciation of the partners' efforts and work, leads to a sense of peace. Appreciation represents paying attention to value and importance of what has happened. When couples ignore their personal wishes because of each other, how beautiful it would be to be appreciated by their partners. No doubt, appreciating any effort leads to repetition of that action and even strengthens that character. In this regard, one participant stated: *“We thank each other even for small things, this is very important, my husband occasionally buy a flower or a little gift for me, this action gives me so much energy and for a few days that flower is in the flower pot, I look at it and remember his goodness, these things are very important.”*

The couple must also pay attention to each others' demands and wishes, and as far as they can, they should try to meet these demands. This demand sometimes is a romantic and purposeful conversation. *“My husband does not have conversation skills, I even suggested to go somewhere together and have a coffee and cake in a coffee shop, we can talk about our problems, for example, I tell you what I except from you and you say what you expect from me, but this stuff looks ridiculous to my husband”.* This was part of a statement made by of one of the participants. Often these demands include financial issues, and for this reason, financial status of men is an influential factor in marital satisfaction. The majority of participants believed that man should be reasonable in terms of economic and social status. Another important influential factor on marital satisfaction according to participants was that, couples should never compare their partners to others in front of the family, friends and acquaintances. This would be considered as a kind of ingratitude and leads to resentment. In regard to this, one of the participants explained: *“When my husband constantly belittle me and compare me with others, I feels humiliated and think I am less than others.”*

Couples' honesty in their behavior and speech and lack of secrecy to one another, creates a sense of peace and increases marital satisfaction. When couples are honest in their words and deeds, they can understand each other´s situations better, and meet the demands of their partners with respect to their abilities. This factor is so important and valuable in life that, one participant said: *“If you take everything from life, maybe the life stay put, but if you take the trust, it will collapse. The best thing about my husband is that, I trust him.”* Therefore, the couples who trust each other and behave in certain way that would foster confidence in the hearts of their partners provide the sense of peace in life for each other.

#### Joyful dependence

Sense of joyful dependence is among senses that increase interest in couples and continuity of their marital lives. This dependence is more beautiful when both spouses have a sense of belonging to each other. One of the participants, who was 25 years old and married for five years, described the feeling like this: *“The most important thing is that, we love each other and our emotional bond is very strong, perhaps it is much more than what can be described, we have an existential dependence, a dependence that I can hold him every day, when I huge him I really feel that, there is nothing else in this world that I want to think about.”*

Another aspect of the couple dependence is the dependent on each other in decision making about life. From the perspective of the participants, the couple should not make decisions for their married life without consulting each other. Marital life as it is obvious from its name is the joint life of the couples, so personal decisions would not be appropriate. This lack of individual decision making helps to create the sense of partnership in all aspects of life.

## Discussion

The results of this these interviews emerged in the form of two themes; maturity of personality and romantic interaction. The theme of maturity of personality included a sub-theme of blossoming of feelings, and the theme of romantic interaction consisted of three sub-themes of; mutual support, sense of peace and joyful dependence. According to the findings, marriage is a process that creates and develops a romantic interaction between couples. In other words, couples entering into marriage will be subject to a process that may lead to the development of their character.

This study showed that, marriage is a process that can transform individuals from depending on parents to being able to make their own decisions in life, this ability includes; ability to gain realistic views of life, ability to be independent and decreased need of getting support from the family, taking responsibility and being committed to them. Consequently, to achieve satisfaction in marital life, the couples have to enrich their abilities of these features. In this regard, several studies have shown that, personality attributes have a great effect on marital satisfaction [21–23]. The participants stated that, marital life gradually teaches people that, until they do not have the ability to compromise with their partner and existing situation, they cannot be satisfied with their lives. Compromising and adjusting with spouse is a process which comes over time with better understanding and knowing of the partner. This feature, along with ignoring some personal desires, and forgiving deficiencies and upsetting issues caused by spouse, family, friends and acquaintances can dramatically increase a sense of satisfaction in people's marital life. This finding is consistent with the findings of studies that have shown, forgiveness has a positive effect on marital satisfaction [24–26]. Chung (2014) in a study of 208 Korean teachers concluded that, increased sense of forgiveness increases the satisfaction and continuation of marital life [27]. Loyalty and commitment in marriage was an important influential factor in marital satisfaction according to the participants in this study. Isanejad et al, also in their study concluded that, loyalty towards spouse can lead to increased sense of marital satisfaction [28].

In our country, having a child is considered a value for couples [29]. However, child birth is an influential phenomenon in marital life. After the birth of the first child, whether the parents wanted to have a child or not, life of the couple and their marital life will be greatly affected, this effect is positive for some couples and negative for others. The result of this study showed that, although the presence of children can reduce couples' time spent with each other, it can also deepen their relationship. However, entry of children cannot have a positive effect on a marriage that has been already in crisis. The closeness of couples' ideas in children´s upbringing is one of the important issues that affect the marital relationship. In this regard, some studies have shown that, parenting skills can increase marital satisfaction [15, 30].

Inducting a sense of loving and being loved, a clear expression of emotions and deep feeling, paying attention to partner's wishes, while complying with mutual respect, and a sense of gratitude, are among influential factors in creating a romantic interaction between married couples. Minnotte, et al study also showed that, emotional contact with spouse and children affects marital satisfaction [31]. Understanding the needs of partner, paying attention to them and trying to meet them, are include those factors that, in different ways, were expressed by the participants in this study. Emotional need, the need for empathy and sexual needs are include the needs that are often ignored by spouses in real life. However, many studies have shown that, efforts to meet those needs are associated with improving marital satisfaction [31–33].

All participants agreed on the importance of trust between couples to achieve marital satisfaction. They believed shortcomings in other aspects of life may be negligible, but when the trust between couples is reduced or eliminated, survival of life would not be possible. Trusting partner had a close relationship with religious and spiritual beliefs. Couples, who knew their husbands as religious persons, had accepted them more in regard to the trust and commitment. They also believed that, having religious beliefs will have a positive impact on them. These findings were consistent with the results of some studies [12, 34, 35].

## Conclusion

Marital life can lead to the maturity of personality and romantic relations between couples. Of course, this requires time and knowing of themselves and their spouses. In fact, the women in this study were satisfied with their marital life when they were able to establish a friendly, supportive and respectful relationship with their husband through the maturity of their personalities. Providers of advisory services to couples need to understand the interaction between wives and husbands. They should know that couples, who can communicate well, can resolve many shortcomings of their marital life. Iranian society is in a transition period from a religious-traditional society to a modern society. Among the main changes in this period, we can point to the increased age of marriage, decreased desire for marriage, high divorce rate, and decreased family stability and marital satisfaction. One of the main reasons for these changes is the lack of young couples' knowledge or changed perspective on philosophy and real meaning of the marriage. In previous generations, religious and traditional teachings and beliefs as well as advice of family elders guided couples to reach marital stability. However, with all the changes happening in today's society and the reduction in religious and tradition's roles, perhaps family advisors play an ever important role in this regard. In comparison with the findings of previous studies, the results of presented study contained valuable concepts such as maturity of personality and romantic interaction which are being referred to less in previous studies. Surely, these concepts in every society have unique meaning. Since, there has not been a qualitative study addressing this issue in Iranian couples, the findings of this study can serve as a guide for family advisors in order to change the perspective of couples in regard to marriage and decrease their expectation and increase their understanding of marital life.

### What is known about this topic

- Marital satisfaction is the feeling of happiness, satisfaction, and joy experienced by the couples;
- Marital satisfaction has an effect on mental and physical health, life satisfaction, success in business and social connections;
- Marital satisfaction is a multidimensional concept that includes various factors such as personality attributes, finances, parenting styles and sexual relations.

### What this study adds

- Marital life can lead to the maturity of personality and to the romantic relations between couples;
- Improve the quality of counseling with couples by increased awareness of family counselors;
- With all the changes happening in today's society and the reduction in religious and tradition's roles, perhaps family advisors should play an ever important role in improvement of marital satisfaction.
